# 肺癌围手术期患者家属焦虑、抑郁状况对患者一般心理状况的影响

**DOI:** 10.3779/j.issn.1009-3419.2011.12.02

**Published:** 2011-12-20

**Authors:** 显宁 吴, 名久 陈, 丹 苏, 风雷 喻

**Affiliations:** 1 410011 长沙，中南大学湘雅二医院心胸外科 Department of Cardio thoracic Surgery, the Second Xiangya Hospital of Central South University, Changsha 410011, China; 2 410013 长沙，中南大学护理学院 Nursing School of Central South University, Changsha 410013, China

**Keywords:** 肺肿瘤, 围手术期, 患者, 家属, 心理, Lung neoplasms, Perioperative period, Patients, Family members, Psychology

## Abstract

**背景与目的:**

面对癌症和手术的双重压力, 许多患者会出现紧张、恐惧、悲观和焦虑等一系列不良心理, 本研究旨在探讨肺癌围手术期患者家属的焦虑、抑郁状况对患者一般心理状况的影响。

**方法:**

以中南大学湘雅二医院普胸外科处于肺癌围手术期的97例患者和97例家属作为研究对象, 采用自制的一般资料问卷、焦虑自评量表(self-rating anxiety scale, SAS)、抑郁自评量表(self-rating depression scale, SDS)以及症状自评量表(symptom check-list 90, SCL-90)对他们的一般资料、肺癌患者家属的焦虑、抑郁状况以及肺癌患者的一般心理状况进行调查。

**结果:**

肺癌围手术期患者SCL-90中总分(153.28±41.98)、躯体化(1.78±0.42)、强迫症状(1.96±0.52)、抑郁(1.77±0.67)、焦虑(1.82±0.56)、敌对(1.68±0.87)、恐怖(1.44±0.75)、精神病性(1.56±0.51)因子分均高于中国常模(*P* < 0.05)。肺癌围手术期患者家属焦虑标准分与患者的SCL-90的强迫症状、焦虑、偏执因子分均呈正相关(*P* < 0.05);肺癌围手术期患者家属抑郁严重度与患者的SCL-90的强迫症状、精神病性因子分均呈负相关(*P* < 0.05)。

**结论:**

肺癌围手术期患者家属存在不同程度的焦虑、抑郁状况, 且对肺癌患者的一般心理状况有一定的影响。

## Abstract (en)

**Background and objective:**

Facing the double pressures caused by cancer and surgery, many patients will appear series psychological problems like nervous, fear, pessimism, anxiety and so on.The aim of this study is to explone the anxiety, depression status of family members, the general psychological status of perioperative patients with lung cancer and the influence of the former on the latter.

**Methods:**

Total 97 patients and 97 patient family members from the thoracic department of the Second Xiangya Hospital of Central South University were enrolled as the study subjects.The general information, the anxiety and depression status of the family members, and the general psychological status of perioperative patients with lung cancer were investigated using self-made general information questionnaire, self-rating anxiety scale (SAS), self-rating depression scale (SDS) and symptom check-list 90 (SCL-90).

**Results:**

The scores of seven factors, including total scores (153.28±41.98), somatization (1.78±0.42), compulsion (1.96±0.52), depression (1.77±0.67), anxiety (1.82±0.56), hostility (1.68±0.87), panic (1.44±0.75) and psychosis (1.56±0.51) in SCL-90 were remarkerbly higher than those in domestic norm (*P* < 0.05).The standard score in SAS of the family members was positively correlated with the compulsion, anxiety and bigoted factors of SCL-90 in the patients (*P* < 0.05).The depression severity of the family members was negatively correlated with the compulsion and psychosis factors of SCL-90 in the patients (*P* < 0.05).

**Conclusion:**

Family members have different degrees of anxiety and depression, which have certain effects on the general psychological status of perioperative patients with lung cancer.

现代医学中手术是治疗肺癌的主要方式, 面对癌症和手术的双重压力, 许多患者会出现紧张、恐惧、悲观和焦虑等一系列不良心理^[1]^, 而这些不良的心理刺激又可导致患者的机体免疫功能降低, 进而引起病情恶化与癌细胞扩散^[2]^。同时, 作为患者直接照顾者的患者家属, 其心理状态直接影响患者心理及病情和转归^[3]^。本文通过对肺癌围手术期患者家属的焦虑、抑郁状况及患者的一般心理状况进行调查, 旨在探讨患者家属的焦虑、抑郁状况对患者一般心理状况的影响。

## 1 对象与方法

### 1.1 对象

本研究采用方便抽样的方法, 于2010年4月-2011年9月从中南大学湘雅二医院普胸外科抽取手术前的肺癌患者及直接承担其照顾任务的家属为调查对象。发放问卷200份, 收回194份, 回收率97.0%。患者纳入标准:经临床、影像学或内镜及病理确诊为非小细胞肺癌(non-small cell lung cancer, NSCLC)患者; 小学及以上文化程度; 经知情同意并签署知情同意书, 自愿加入研究项目; 意识清楚, 能正确表达自己的意愿。家属纳入标准:小学及以上文化程度; > 18岁; 经知情同意并签署知情同意书, 自愿加入研究项目; 意识清楚, 能正确表达自己的意愿。本研究97例肺癌患者中, 男性73例(75.3%), 女性24例(24.7%); 年龄30岁-67岁, 平均年龄50.6岁; 97例肺癌患者家属中, 男性61例(62.9%), 女性36例(37.1%); 年龄20岁-63岁, 平均39.1岁。

### 1.2 工具

#### 1.2.1 自制的一般资料问卷

包括年龄、性别、民族、文化程度、婚姻状况、医疗费用支付方式及宗教信仰等。

#### 1.2.2 焦虑自评量表(self-rating anxiety scale, SAS)^[4]^

SAS含有20个条目, 采用1级-4级评分。将20个项目的各个得分相加, 得到粗分; 用粗分乘以1.25后取整数部分, 就得到标准分, 按照中国常模(即中国成人的一般标准)结果, SAS标准分的分界值为50分, 其中50分-59分为轻度焦虑, 60分-69分为中度焦虑, ≥70分为重度焦虑。

#### 1.2.3 抑郁自评量表(self-rating depression scale, SDS)^[4]^

由20个条目组成, 按1级-4级评分。20个条目反映抑郁状态四组特异性症状。抑郁严重度指数=各条目累计分/80。 < 0.5者为无抑郁; 0.5-0.59为轻微-轻度抑郁; 0.6-0.69为中-重度; ≥0.7为重度抑郁。

#### 1.2.4 症状自评量表(symptom check-list 90, SCL-90)^[4]^

本测验共90个自我评定项目。测验的9个因子分别为:躯体化、强迫症状、人际关系敏感、抑郁、焦虑、敌对、恐怖、偏执及精神病性。每一个项目均采取1级-5级评分制。

上述量表均有良好的信度和效度。

### 1.3 统计学方法

采用SPSS 13.0软件进行数据处理, 采用单样本*t*检验、单因素方差分析、*Pearson*相关分析、多元线性回归分析等, 方法分析数据以*P* < 0.05为差异有统计学意义。

## 2 结果

### 2.1 肺癌围手术期患者家属SAS、SDS调查结果

97例肺癌围手术期患者家属焦虑标准分为35分-61分[平均(47.50±6.59)分], < 50分者(无焦虑)62例, 占63.9%, 50分-59分之间者(轻度焦虑者)29例, 占29.9%, 60分-69分之间者(中度焦虑者)6例, 占6.2%;抑郁标准分为38分-59分[平均(51.33±5.27)分], 抑郁严重度为0.38-0.59(平均0.51±0.05), 其中无抑郁组19例(19.6%), 轻微-轻度抑郁组78例(80.4%)。

### 2.2 肺癌围手术期患者SCL-90调查结果

肺癌围手术期患者SCL-90中总分、躯体化、强迫症状、抑郁、焦虑、敌对、恐怖、精神病性因子分均高于中国常模^[5]^(*P* < 0.05)。见表 1。

**1:** 肺癌围手术期患者SCL-90与常模评分比较 Compared SCL-90 scores of perioperative patients with lung cancer to domestic norm

Factors	Patients (*n*=97)	Norm^[5]^ (*n*=1, 388)	*t*	*P*
Total scores	153.28±41.98	129.96±38.76	5.47	< 0.001
Somatisation	1.78±0.42	1.37±0.48	9.59	< 0.001
Compulsion	1.96±0.52	1.62±0.58	6.42	< 0.001
Interpersonal sensitivity	1.66±0.68	1.65±0.61	0.11	0.914
Depression	1.77±0.67	1.50±0.59	3.99	< 0.001
Anxiety	1.82±0.56	1.39±0.43	7.46	< 0.001
Hostility	1.68±0.87	1.46±0.55	2.49	0.014
Panic	1.44±0.75	1.23±0.41	2.77	0.007
Bigoted	1.41±0.38	1.43±0.57	-0.61	0.546
Psychosis	1.56±0.51	1.29±0.42	5.18	< 0.001

### 2.3 肺癌围手术期患者家属焦虑、抑郁状况与患者一般心理状况的相关分析

肺癌围手术期患者家属焦虑标准分与患者的SCL-90的强迫症状、焦虑、偏执因子分均呈正相关(*P* < 0.05);肺癌围手术期患者家属抑郁严重度与患者的SCL-90的强迫症状、精神病性因子分均呈负相关(*P* < 0.05)。见表 2。

**2:** 肺癌围手术期患者家属焦虑、抑郁状况与患者一般心理状况的相关性 Correlations between the anxiety and depression status of family members and the general psychological status of perioperative patients with lung cancer

Factors	SCL-90 total scores	Compulsion	Depression	Anxiety	Panic	Bigoted	Psychosis
The standard score in SAS	0.188	0.238^*^	0.121	0.216^*^	0.053	0.247^*^	0.078
The depression severity	-0.149	-0.297^#^	-0.086	-0.014	-0.123	-0.176	-0.231^*^
SDS:self-rating depression scale; SCL-90:symptom check-list 90;^*^P < 0.05;^#^*P* < 0.01.							

### 2.4 肺癌围手术期患者家属焦虑、抑郁状况对患者一般心理状况的回归分析

根据相关分析的结果, 以肺癌围手术期患者家属焦虑标准分、抑郁严重度为自变量, 分别以强迫症状、焦虑、偏执、精神病性各因子为因变量进行四次多元线性回归分析, 以强迫症状因子为因变量的多元线性回归结果显示2个自变量均进入了回归等式, 且决定系数为0.148, 表明肺癌围手术期患者强迫症状因子的14.8%可由此2个变量的变化来解释; 以焦虑因子为因变量的多元线性回归结果显示仅焦虑标准分进入了回归方程, 且可解释总变异的4.7%;以偏执因子为因变量的多元线性回归结果显示仅焦虑标准分可以进入回归等式, 且决定系数为0.094, 说明患者偏执因子的9.4%可以由家属的焦虑标准分的变化来解释; 以精神病性因子为因变量的多元线性回归结果显示仅抑郁严重度可以进入回归等式, 且决定系数为0.060, 可解释总变异的6.0%。见表 3。

**3:** 患者一般心理状况的回归分析 Regression analysis to the general psychological status of perioperative patients with lung cancer

Dependent variable	Independent variable	B	Beta	*t*	*P*	*R*^2^
Compulsion	The standard score in SAS	0.020	0.246	2.582	0.011	0.148
	The depression severity	-3.056	-0.303	-3.187	0.002	
Anxiety	The standard score in SAS	0.020	0.217	2.152	0.034	0.047
	The depression severity	-0.222	-0.020	-0.201	0.841	
Bigoted	The standard score in SAS	0.015	0.252	2.568	0.012	0.094
	The depression severity	-1.358	-0.183	-1.861	0.066	
Psychosis	The standard score in SAS	0.007	0.085	0.845	0.400	0.060
	The depression severity	-2.308	-0.233	-2.331	0.022	

## 3 讨论

当肺癌患者认识到自己的病情且手术要切除肺叶时, 常出现焦虑、抑郁、恐惧等异常情绪, 特别是初次手术的患者焦虑程度较高^[6]^。Ramsay等^[7]^进行的一项多达382例术前患者的调查分析显示:73%的术前患者有恐惧感, 62%的术前患者害怕麻醉, 45%的术前患者害怕手术, 23%的术前患者害怕术后不能苏醒, 16%的术前患者担心术中出现疼痛, 5%的术前患者害怕术后疼痛。由此可见, 多数手术的患者在术前就有精神紧张、焦虑、恐惧以及夜间失眠等情绪反应。本研究中肺癌围手术期患者SCL-90得分与常模的比较结果可以说明此状况。研究^[8]^表明, 紧张状态有刺激肿瘤发展和生长的作用, 不良心理会加重治疗的不良反应, 极大地影响癌症患者手术后的生活质量。可见, 关注肺癌围手术期患者的心理状况意义重大。

肺癌患者的心理状况与多方面因素有关, 他们不仅要面临肺癌本身作为恶性肿瘤带来的压力, 还要面临手术这一负性生活事件带来的心理负担, 除此之外, 肺癌患者家属的影响也应受到人们的关注。患者家属的焦虑、抑郁状况会对患者的一般心理状况产生影响, 本研究结果中患者家属的焦虑标准分、抑郁严重程度与患者SCL-90的多个因子存在相关性, 且能够进入以强迫症状、焦虑、偏执、精神病性各因子为因变量的多元线性回归方程, 可以充分的说明此问题。由此可见, 家属的焦虑、抑郁程度不仅会影响其自身的身心健康, 同时对患者的心理、治疗乃至以后的康复都会产生很大的负面作用, 我们在进行心理治疗时不能忽略家属的心理反应, 要对患者及其家属同时进行心理干预方可达到理想效果。

综上所述, 我们在重视肺癌围手术期患者的手术及药物治疗的同时, 也不能忽略其心理问题, 要达到理想的身心治疗效果, 就要对肺癌患者及其家属的心理状况有充分的研究并知道他们的心理状况之间存在相关性, 从而制定出有针对性的全面的心理干预措施。
